# Pregnancy Differentially Impacts Performance of Latent Tuberculosis Diagnostics in a High-Burden Setting

**DOI:** 10.1371/journal.pone.0092308

**Published:** 2014-03-21

**Authors:** Jyoti S. Mathad, Ramesh Bhosale, Vikrant Sangar, Vidya Mave, Nikhil Gupte, Savita Kanade, Ashwini Nangude, Kavita Chopade, Nishi Suryavanshi, Prasad Deshpande, Vandana Kulkarni, Marshall J. Glesby, Daniel Fitzgerald, Renu Bharadwaj, Pradeep Sambarey, Amita Gupta

**Affiliations:** 1 Division of Infectious Diseases, Weill Cornell Medical College, New York, New York, United States of America; 2 Department of Obstetrics and Gynaecology, Byramjee Jeejeebhoy Government Medical College- Sassoon General Hospital, Pune, Maharashtra, India; 3 Byramjee Jeejeebhoy Government Medical College- Johns Hopkins Clinical Trials Unit, Pune, Maharashtra, India; 4 Department of Microbiology, Byramjee Jeejeebhoy Government Medical College- Sassoon General Hospital, Pune, Maharashtra, India; 5 Division of Infectious Diseases, Johns Hopkins University School of Medicine, Baltimore, Maryland, United States of America; University of Cape Town Lung Institute, South Africa

## Abstract

**Background:**

Targeted screening for latent TB infection (LTBI) in vulnerable populations is a recommended TB control strategy. Pregnant women are at high risk for developing TB and likely to access healthcare, making pregnancy an important screening opportunity in developing countries. The sensitivity of the widely-used tuberculin skin test (TST), however, may be reduced during pregnancy.

**Methods:**

We performed a cross-sectional study comparing the TST with the QuantiFERON Gold In-tube (QGIT) in 401 HIV-negative women presenting antepartum (n = 154), at delivery (n = 148), or postpartum (n = 99) to a government hospital in Pune, India. A subset of 60 women enrolled during pregnancy was followed longitudinally and received both tests at all three stages of pregnancy.

**Results:**

The QGIT returned significantly more positive results than the TST. Of the 401 women in the cross-sectional study, 150 (37%) had a positive QGIT, compared to 59 (14%) for the TST (p<0.005). Forty-nine (12%) did not have their TST read. Of 356 who had both results available, 46 (13%) were concordant positive, 91 (25%) were discordant (12 (3%) TST+/QGIT-; 79 (22%) TST−/QGIT+), and 206 (57%) concordant negative. Comparison by stage of pregnancy revealed that QGIT percent positivity remained stable between antepartum and delivery, unlike TST results (QGIT 31–32% vs TST 11–17%). Median IFN-γ concentration was lower at delivery than in antepartum or postpartum (1.66 vs 2.65 vs 8.99 IU/mL, p = 0.001). During postpartum, both tests had significantly increased positives (QGIT 31% vs 32% vs 52%, p = 0.01; TST 17% vs 11% vs 25%, p<0.005). The same trends were observed in the longitudinal subset.

**Conclusions:**

Timing and choice of LTBI test during pregnancy impact results. QGIT was more stable and more closely approximated the LTBI prevalence in India. But pregnancy stage clearly affects both tests, raising important questions about how the complex immune changes brought on by pregnancy may impact LTBI screening.

## Introduction

Globally, 800 million women carry latent *Mycobacterium tuberculosis* infection (LTBI), and 3 million develop active TB disease every year. TB disproportionately affects women between the ages of 15 and 45 [1], when they are most likely to become pregnant. The disease is twice as likely to reactivate postpartum than at any other time in a woman's life [2]. A woman whose LTBI reactivates during pregnancy has a high risk of death, prenatal complications and poor fetal outcomes[3]–[5].

Models show that treating LTBI is essential to slowing the spread of disease [6]. With nearly 180 million pregnancies occurring annually in moderate/high TB burden settings, and up to 40% of these women having LTBI, pregnancy is an ideal time to focus on TB prevention strategies, including isoniazid preventive therapy (IPT). The challenge is identifying pregnant women carrying the infection.

There are two common screening tests. The tuberculin skin test (TST) is inexpensive and requires no laboratory infrastructure, but has low specificity [7] and requires the patient to return in 48–72 hours—a challenge for many. The newer interferon gamma release assay (IGRA) test uses a blood draw. While the IGRA is more specific and eliminates the need for a return visit, the test is more expensive and requires laboratory infrastructure lacking in many hospitals in developing countries. If, however, the IGRA were proven significantly more reliable than the TST, especially in key populations such as pregnant women, there would be a compelling argument to consider it for targeted LTBI screening and IPT administration to prevent maternal-child TB.

There is a long-held suspicion that pregnancy-induced immune suppression affects LTBI screening tests, but studies on the relative performance of the tests in pregnancy come exclusively from low TB burden countries and show divergent results [8]–[12]. There are no studies comparing the performance of both tests in pregnant women in a high- TB burden country.

The objectives of our study were to examine how pregnancy impacts the performance of LTBI diagnostics and to establish the concordance of the QuantiFERON TB Gold In-tube Test (QGIT), an IGRA, with the TST during pregnancy and the postpartum period in a TB-endemic country.

## Methods

### Ethics Statement

This study was approved by the Johns Hopkins University Institutional Review Board (IRB), the Weill Cornell Medical College IRB, the Byramjee Jeejeebhoy Government Medical College IRB and the Byramjee Jeejeebhoy Government Medical College Ethics Committee in India. All subjects provided written informed consent.

### Study Population

From January 2011 through July 2012, we prospectively enrolled HIV-negative women presenting to the antenatal clinic (ANC) in their late second or third trimester, to the delivery ward, or to the pediatric immunization clinic at least 3 months postpartum into a cross-sectional study at Sassoon General Hospital (a public teaching hospital affiliated with Byramjee Jeejeebhoy Government Medical College (BJGMC)) in Pune, India. Each weekday (Monday-Friday) morning, women presenting to each location were approached for enrollment. After the first 154 consented, no additional women were screened. Enrollment of antepartum women was complete within 4 months; enrollment of eligible women at delivery and postpartum was more difficult and took 12–14 months to complete. To evaluate the outcome of serial testing, 60 antepartum women were followed longitudinally with repeat testing planned at delivery and postpartum. All participants were at least 18 years old. Women with a history of allergic reaction to the TST, current active TB, or an immunosuppressive condition were excluded. Women not tested for HIV since becoming pregnant were offered HIV testing and excluded if HIV-positive. Women discharged within 24 hours of delivery were excluded.

### Questionnaires

Trained counselors administered sociodemographic questionnaires, including the Household Food Insecurity Access Scale (HFIAS) [13]. Trained nurses obtained medical and obstetric histories, including information about TB risk factors and a TB symptom screen (i.e. cough, fever, night sweats, or weight loss/ lack of appropriate weight gain of any duration). Women with positive symptom screens were referred to a physician for formal evaluation. If active TB was excluded, the subject was eligible for enrollment.

### Testing for LTBI

Trained lab staff obtained 3 mL of blood per enrollee for QuantiFERON Gold Test-in-tube (QGIT) testing; 1 mL each for the negative control tube, positive mitogen control tube and TB-specific antigen tube. The test was performed in accordance with the manufacturer's package insert [14] at a laboratory certified by the College of American Pathologists (CAP) to perform QGIT testing. Blood samples were incubated at 37°C for 16–24 hours. After incubation, the tubes were centrifuged, plasma was removed and ELISA was used to measure the interferon (IFN)-γ concentrations. If the difference in IFN-γ concentrations between the TB antigen tube and nil tube was ≥0.35 IU/mL and ≥25% of the concentration from the nil tube, it was considered to be positive. A sample was recorded as indeterminate if the difference in IFN-γ concentration between the mitogen tube and nil tube was <0.5 IU/mL or if the nil tube concentration was >8 IU/mL [14]. If a QGIT result was indeterminate, the sample was repeated. If the repeat sample was indeterminate again, it was recorded as such. If, however, the sample was positive or negative, the result of the second sample was recorded.

After phlebotomy for QGIT, trained nurses injected 0.1 mL (5TU) of tuberculin PPD intradermally into the volar surface of the forearm. Outpatients were asked to return in 48–72 hours for interpretation of the TST and a compensation of INR 100 (∼$2 USD) was given to cover travel costs. For women enrolled at delivery, attempts were made to interpret the TST before hospital discharge. A positive TST was defined as induration ≥10 mm.

For the longitudinal cohort, TST and QGIT were performed as described above at antepartum, and repeated again at delivery and postpartum. We defined a transition from a negative TST to a positive TST as a baseline TST <10 mm with follow-up TST of ≥10 mm, with an increment of at least 10 mm [15]. For the QGIT, this was defined as a baseline negative test followed by a positive one. Transition from a positive to a negative test for both tests was defined as a previous positive test followed by a negative one.

### Sample Size Calculation and Statistical Analysis

The kappa statistic was used to quantify the degree of agreement between the QGIT and TST. With our sample size, a two-sided 95% confidence interval for the kappa statistic would extend < = 0.17 from the observed value of kappa, assuming that the true value of kappa is in the range 0.50–0.70 and the approximate prevalence of latent TB is 20–30%. For comparison of categorical variables (e.g. residence, employment), the Fisher's exact test was used. The Wilcoxon ranksum test was used for comparison of continuous variables. All p-values were two-sided with statistical significance evaluated at the 0.05 alpha level. Risk factors for test positivity and test discordance (e.g. positive TST/negative QGIT or negative TST/ positive QGIT) were calculated using a logistic regression model. From this, odds ratios (OR) with 95% confidence intervals (CI) were determined. Variables that were statistically significant or had a trend towards significance (p <0.2) in univariate analysis or were of clinical importance were included in the multivariate analysis. All data was entered into an onsite Access database, and analyses were performed in Stata Version 12.0 (StataCorp, College Station, Texas).

## Results

### Population characteristics:

We enrolled 154 antepartum women, 148 women at delivery, and 99 postpartum women. Their baseline characteristics are described in (**Table 1**). The median gestational age in the antepartum group was 27 weeks (IQR 23–30) and the median postpartum time was 12 weeks (IQR 11–15). Of all participants, one antepartum woman reported a personal history of TB and one postpartum woman had received IPT in the past. Ten (2.4%) women reported a known TB contact.

**Table 1:** Participant characteristics and LTBI test results by time point of screening in Pune, India.

Characteristic^a^	Antepartum (n = 154)	Delivery (n = 148)	Postpartum (n = 99)	p value
*Sociodemographic factors (household)*				
Urban/periurban residence	149 (96%)	122 (82%)	95 (95%)	<0.005
House with ≤2 rooms^b^	124 (80%)	104 (70%)	79 (79%)	0.05
Joint family type	111 (72%)	86 (58%)	67 (67%)	0.05
Median Adults in house (IQR)	5 (3–6)	4 (2–5)	4 (2–6)	0.006
Median children in house (IQR)	1 (1–2)	2 (1–2)	2 (1–3)	0.06
*Sociodemographic factors (individual)*				
Employed for pay	13 (8.3%)	11 (7.3%)	4 (3%)	0.39
Education level ≤4^th^ grade	19 (12%)	24 (16%)	6 (6%)	0.05
Biomass Cooking Fuel	12 (7.7%)	22 (14%)	5 (5%)	0.009
Moderate to severe food insecurity^c^	16 (10%)	6 (3.9%)	12 (12%)	0.02
*Obstetric History*				
Median gestational age, wks (IQR)	27 (23–30)	NA	NA	NA
Median postpartum time, wks (IQR)	NA	NA^d^	12 (11–15)	NA
First prenatal visit	16 (10%)	NA	NA	NA
First pregnancy	64 (41%)	69 (46%)	43 (43%)	0.67
*Tuberculosis Risk Factors (individual)*				
Chest infection in past year	1 (0.6%)	0 (0%)	0 (0%)	0.44
Treated for TB >30d	1 (0.6%)	0 (0%)	0 (0%)	0.44
History of IPT	0 (0%)	0 (0%)	1 (1%)	0.22
Close contact diagnosed or treated for TB	6 (3.8%)	2 (1.3%)	2 (2%)	0.34
Contact with MDR-TB	1 (0.6%)	1 (0.6%)	1 (1%)	0.94
Positive TB symptom screen^e^	12 (7.4%)	16 (10%)	1 (1%)	0.01
*Tuberculosis Risk Factors (household)*				
Household contact TB symptoms^e^	29 (18%)	5 (3.3%)	7 (7%)	<0.05
Smoker in the house	27 (17%)	23 (15%)	20 (20%)	0.63
*LTBI test results*				
**TST**				<0.005
Positive	25 (16%)	16 (10%)	18 (18%)	
*Median induration, mm (IQR)*	15 (10–20)	13 (10–16)	14 (12–15)	0.44
Negative	115 (74%)	126 (85%)	52 (52%)	
Did not return	14 (9%)	6 (4%)	29 (29%)	
**QGIT**				0.01
Positive	50 (32%)	48 (32%)	52 (52%)	
*Median IFN-γ, IU/mL (IQR)*	2.65 (1.17–5.56)	1.66 (0.77–3.16)	8.99 (1.92–10)	0.001
Negative	99 (64%)	96 (64%)	47 (47%)	
Indeterminate	5 (3.2%)	4 (2.7%)	0 (0%)	

a Missing variables not included in calculations.

b Excluding kitchen and bathroom.

c Household Food Insecurity Access Scale: Category 1 =  Food Secure, Category 2 =  Mildly Food Insecure, Category 3 =  Moderately Food Insecure, Category 4 =  Severely Food Insecure.

d All women enrolled within 24–48 hours of delivery.

e TB symptom screen is positive if cough, fever, weight loss, or night sweats are present.

Abbreviations: HIV indicates human immunodeficiency virus, IPT indicates isoniazid preventive therapy, IQR indicates interquartile range, MDR-TB indicates multi-drug resistant tuberculosis, NA indicates not applicable, TB indicates tuberculosis, TST indicates tuberculin skin test, QGIT indicates QuantiFERON-TB Gold Test In-Tube.

### Comparison of TST and QGIT performance

**Figure 1:**
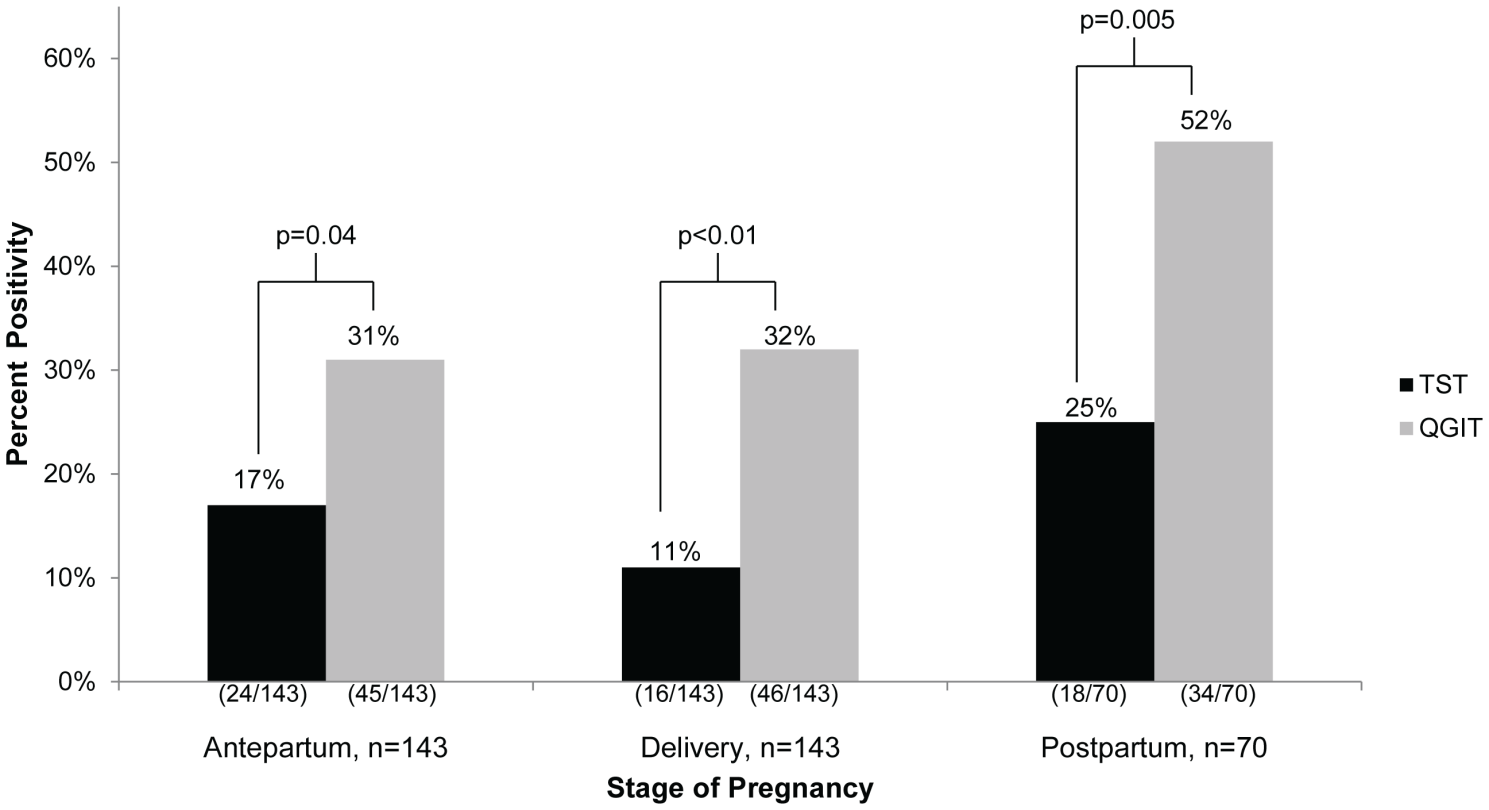
Cross-sectional comparison of TST and QGIT positivity by stage of pregnancy^a^. QGIT positivity was significantly higher than TST positivity at each stage of pregnancy. TST positivity was lowest during delivery and highest in postpartum women. QGIT positivity was stable during antepartum and delivery but was also higher in postpartum women. There was a trend towards a significant difference in TST positivity between antepartum versus delivery (p = 0.17) and antepartum versus postpartum (0.20), and a significant difference between delivery versus postpartum (0.009). There was no significant difference in QGIT positivity between antepartum versus delivery (p = 0.89), but there was a trend towards significance between antepartum versus postpartum (0.11) and a significant difference between delivery and postpartum (p = 0.02). ^a^The number of women who did not return for TST reading was 11 from antenatal, 5 from delivery and 29 from postpartum. Results shown here only include women with both TST and QGIT results. Abbreviations: QGIT =  QuantiFERON TB Gold In-tube Test; TST =  tuberculin skin test.

**Figure 2:**
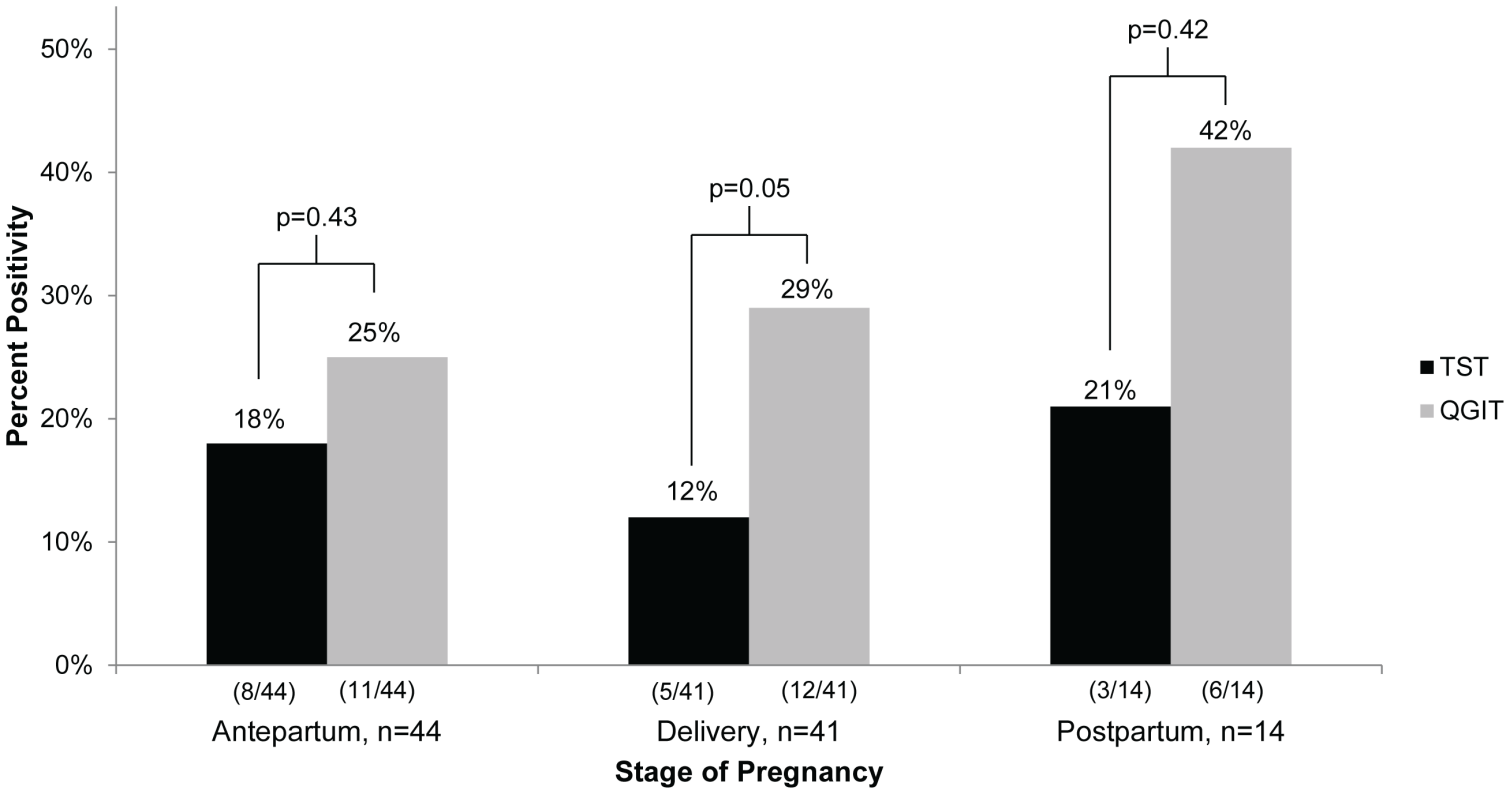
Longitudinal comparison of TST and QGIT positivity by stage of pregnancy^a^. QGIT positivity was higher than TST positivity at each stage of pregnancy, but only reached statistical significance at delivery. TST positivity was lowest during delivery and highest in postpartum women. QGIT positivity was also highest in postpartum women. ^a^Includes results for women who had TST and QGIT test results for at least 2 different visits: antepartum/delivery, delivery/postpartum, or antepartum/postpartum. Abbreviations: QGIT =  QuantiFERON TB Gold In-tube Test; TST =  tuberculin skin test.

Overall, 37% of women had a positive QGIT and 14% had a positive TST (p<0.005). Among the 356 (88%) women who had both a QGIT and TST result available for analysis, the proportion positive by each of the two tests was statistically different in each pregnancy stage group: antepartum (32% QGIT vs. 18% TST, p = 0.03), at delivery (31% QGIT vs. 11% TST, p<0.01) and postpartum (48% QGIT vs. 25% TST, p = 0.005) (**Figure 1**). The same trend was seen in the longitudinal cohort, shown in **Figure 2**. Among women who had TST and QGIT results for more than one stage of pregnancy, TST positivity went from 19% antepartum to 12% at delivery, and back to 18% postpartum. QGIT positivity remained more stable at 26% antepartum and 29% at delivery with a higher percentage of women (45%) positive postpartum.

Among the 45 women who did not return for TST reading, 30 (66%) were QGIT positive. All five antepartum women with indeterminate QGIT results had a negative TST. In the delivery group, three of the four women with indeterminate QGIT were TST negative and one was TST positive (induration  = 17 mm) (**Table S1a–c**).

The lone antepartum woman who reported a history of TB was TST negative and QGIT positive. Nine of 10 (90%) women who had contact with someone with TB had a negative TST (1 did not return for TST reading) but 5 (50%) had a positive QGIT.

### Concordance and discordance

**Figure 3:**
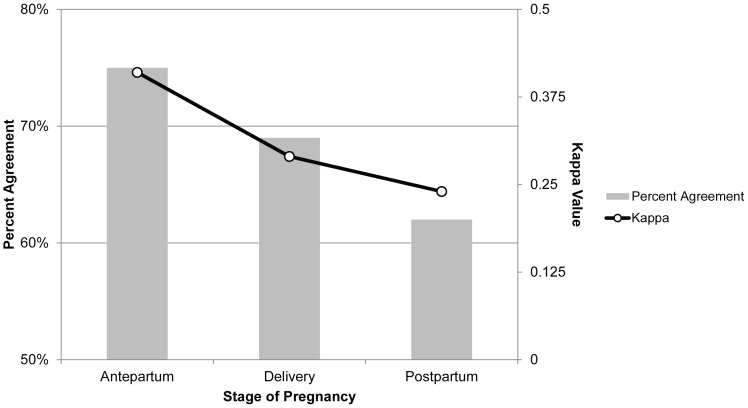
Agreement between TST and QGIT decreases by stage of pregnancy. Percent agreement and kappa were highest in antepartum women and lowest in postpartum women. Abbreviations: TST indicates tuberculin skin test, QGIT indicates QuantiFERON-TB Gold Test In-Tube.

The overall agreement between TST and QGIT was 76% (κ = 0.37); it was 75% (κ = 0.41) antepartum, 69% (κ = 0.29) at delivery and 62% (κ = 0.24) postpartum (**Figure 3**). A total of 20 (12%) antepartum women, 13 (8%) women at delivery, and 13 (13%) postpartum women had concordant positive TST and QGIT. Decreasing the cutoff of the TST or the QGIT did not significantly affect concordance (**Table S2a–b**).

There was a similar prevalence of discordance in both antepartum and delivery groups (21% vs. 24%, respectively) but higher discordance in the postpartum group (37%, p = 0.04). Positive TST/ negative QGIT results were observed in 5 (3%) antepartum women and 5 (5%) postpartum women, but only 2 (1%) at delivery (p = 0.09). In comparison, negative TST/ positive QGIT results were more common in all groups, with a slightly lower proportion in the antepartum women as compared to the delivery and postpartum groups (16% vs. 22% vs 21%, respectively; p = 0.13).

In multivariate analysis adjusting for stage of pregnancy, employment, positive TB symptom screen, and known TB contact, being employed (OR 3.0, 95% CI 1.2–7.5, p = 0.01) and postpartum enrollment (OR 2.6, 95% CI 1.3–5.1, p = 0.004) were significantly associated with discordance.

### Influence of pregnancy stage

#### TST

**Table 1** shows the cross-sectional percent positivity of TST by stage of pregnancy. Overall, 16% (95% CI 10–23%) were positive in the antepartum group versus 10% (95% CI 6–16%) at delivery and 18% (95%CI 11–27%) in the postpartum group (p = 0.18). The median induration for a positive TST was similar (13–15 mm, p = 0.4) between the three groups. Of the antepartum women, 14 (9%) did not return for TST reading versus 6 (4%) in the delivery group and 29 (29%) in the postpartum group (p<0.005).

In multivariate analysis adjusting for stage of pregnancy, location of residence, education, employment, religion and family type, a positive TST was significantly associated with education below 4^th^ grade (OR 3.1, 95% CI 1.4–6.8, p = 0.005) and there was a trend associated with being postpartum (OR 1.9, 95% CI 0.9–3.9, p = 0.07) (**Table S3**).

#### QGIT

All 401 (100%) women enrolled had QGIT results available for analysis. Percent positivity of the QGIT for each cross-sectional group is also shown in **Table 1**, with 32% (95% CI 25–41%) positive among antepartum, 32% (95% CI (24–40%) at delivery, and 52% (95% CI 42–62%) among postpartum. Four (2.5%) antepartum women and four (2.6%) women at delivery had indeterminate QGIT results. Among those with a positive QGIT, the median concentration of the IFN-γ changed significantly based on stage of pregnancy: 2.6 IU/mL (IQR 1.23–5.64) antepartum, 1.66 IU/mL (IQR 0.80–3.14) at delivery, and 8.9 IU/mL (IQR 2.9–10) postpartum (p = 0.0001).

In multivariate analysis adjusting for stage of pregnancy, location of residence, employment, religion and family type, having an urban/periurban residence (OR 2.5, 95% CI 1.0–5.9, p = 0.03) and being postpartum (OR 2.3, 95% CI 1.3–3.9, p = 0.002) were significantly associated with a positive QGIT (**Table S3**).

### Longitudinal follow-up

Among the 60 women followed longitudinally, more women changed (negative to positive result) with the QGIT versus the TST (12% vs 7%), especially between delivery and postpartum where 5 (50%) of the 10 women with a negative QGIT at delivery had a positive QGIT postpartum. On the other hand, more women changed (positive to negative) with the TST versus QGIT(11% vs. 6%), especially between antepartum and delivery: 5 (62%) of 8 women with a positive TST antepartum had a negative TST at delivery (**Table S4**).

## Discussion

Our study is the first to document how stage of pregnancy influences LTBI diagnostics in a TB-endemic country. The results indicate that reliance on the TST during pregnancy may underestimate the burden of LTBI. The TST results suggest that just 14% of study participants carry LTBI, a number less than half of the estimated 35–40% LTBI prevalence in India [16]. The QGIT returned a more likely positivity rate of 37%. QGIT positivity was also associated with living in urban areas, a known risk factor for TB transmission. Moreover, women with a history of TB or known TB contacts—the patients most likely to have LTBI—were more likely to test positive using the QGIT than using the TST.

The type of discordance that dominated in our study, QGIT positive/TST negative, has not previously been observed among pregnant women [10]–[12]. Discordance was associated with employment outside the house, which increases the risk of new TB exposures. It was also associated with postpartum testing. We believe that the explanation for the disparate results lies in the immune changes associated with pregnancy, observed most clearly in the variability of test results between the antepartum and postpartum periods.

Increasing levels of progesterone in pregnancy favor a Th2-type immune response, suppressing the cell-mediated Th1 immune response [17], which must be intact for both the TST and the QGIT to function properly. There is reason to believe, however, that the impact of cell-mediated immune suppression on the two tests is unequal. The TST requires a fully functioning Th1 system in order to trigger the delayed-type hypersensitivity reaction, which causes skin induration [18], [19]. (This response decreases with other causes of Th1 suppression, like age [20], [21] and immune compromise [22], including steroid use [23].) The QGIT likewise relies on Th1 immune function, but it only measures stimulated concentrations of IFN-γ from the blood ex-vivo, using ELISA. Deficiencies in other Th1 cytokines, such as IL-2 and TNF-α, would not affect QGIT results. Another potential explanation of the discordance involves which cells are stimulated in each test. The ex-vivo secretion of IFN-γ in the QGIT reflects MTB-specific effector T cell function, which requires persistent antigenic stimulation [24]. The TST, on the other hand, requires both effector and central memory T cells, the latter of which may be more vulnerable to suppression by the increased numbers of T regulatory cells [19] during pregnancy.

Th1 suppression reaches its nadir in the late second and third trimesters of pregnancy [25], [26], which could explain why women in the study by Worjoloh, et al, who were mainly in their first trimester, did not exhibit this type of discordance [10]. It could also indicate why, in our study, the median TST induration and IFN-γ concentration were lower at delivery than antepartum. Interestingly, Lighter-Fisher, et al., conducted serial IGRA testing in 25 pregnant women in the US and also noted a decrease in IFN-γ between the first and third trimesters, though it did not reach statistical significance [12].

Th1 cell-mediated immunity recovers dramatically in the postpartum period, similar to an immune reconstitution syndrome [26]. The swing back toward Th1 predominance likely explains why more women tested positive postpartum than at any other period using either the TST or QGIT. Other studies have detected subtle indicators of the same effect: A study in Kenya, for example, found that IFN-γ responses increased in postpartum HIV-infected women up to one year post delivery [27]. It may also account for the fact that postpartum women are at the highest risk of developing and being diagnosed with active TB [2].

The majority of women in the delivery group had Caesarean sections, which may complicate the measurement of Th1 function/cytokines. If this were an issue, however, we would have expected a higher IFN-γ level from the negative control (nil) tubes at delivery versus the other time points, which we did not observe [data not shown]. Moreover, the main difference in test performance was between antepartum/delivery and postpartum, which would not be affected by type of delivery.

It is unlikely that previous TSTs contributed to boosting of either TST or QGIT in the postpartum period, though the smaller sample size of the longitudinal cohort prevents a firm conclusion. Notably, there were no significant differences in test positivity between the cross-sectional and longitudinal subjects at each stage of pregnancy. TST boosting is maximal at 1–4 weeks post initial TST and minimal after 60 days [28], which is when most of our women came for follow-up. There is less of a consensus on IGRA boosting. A meta-analysis concluded that IGRA boosting can occur after TST in 2–12% of people, but most who experienced it were TST positive at baseline [29], which was not the case in our population. More recent studies minimize the possibility of IGRA boosting [30], [31].

Similar to other studies of LTBI diagnostics, we faced the challenge of having no gold standard for LTBI diagnosis. We used TST for comparison, as it is the most widely used LTBI screening test. We continue to contact our study subjects by phone to ascertain incident cases of active TB to assess the predictive value of both tests. However, other studies of pregnant women from TB-endemic countries show that a positive IGRA is associated with an increased risk of developing active TB [32].

We had difficulty retaining women in postpartum care, which reduced our sample size in the longitudinal cohort. Despite the smaller sample size, the trends of QGIT and TST positivity by stage of pregnancy in the longitudinal group were similar to those in the larger cross-sectional cohort. Moreover, the populations were relatively homogenous with respect to latent TB risk factors. Differences in test performance are likely to be related to the tests rather than the population. More than 10% of women did not return for their TST result, especially in the postpartum group. This is similar to what other studies have observed [10], [33] and highlights one of the weaknesses of TST.

Despite these limitations, we observed compelling changes in positivity rates associated with stage of pregnancy. The effect was especially pronounced with the TST. Importantly, this study exposes gaps in our understanding of the fluctuations in immune function during pregnancy and its effects on immune-based tests and infections like TB.

### Implications

Pregnancy represents an opportunity to address TB in India and other resource-poor countries, where institutional, educational, economic, and cultural barriers prevent many women from accessing healthcare during much of their lives [34]. Over 75% of Indian women, however, receive antenatal care, mostly at health care facilities [35]. It is essential to leverage this rare interaction with the healthcare system to optimize preventive health strategies for pregnant women, including TB prevention.

LTBI treatment is a valuable but underutilized TB prevention strategy in high-risk pregnant women. HIV-negative individuals with a positive LTBI test have a 10% lifetime risk of developing active TB [36]. Women have the highest incidence of TB during their reproductive years and are most likely to develop active TB postpartum [1], [2]. In those with a positive LTBI test, IPT can reduce the risk of developing active TB by as much as 60% [36]. Because pregnant women attend multiple antenatal visits, treatment of LTBI during pregnancy also allows close monitoring for potential adverse reactions.

The most recent World Health Organization (WHO) recommendations state that TB-endemic countries should continue to use the TST for LTBI screening, because it performs similarly to IGRAs and is less expensive [37]. In our study, however, 4–29% of women did not return for TST reading, the majority of whom were QGIT-positive. This suggests that the TST may not be appropriate for targeted screening of pregnant and postpartum women in high TB burden countries for both operational and immunologic reasons.

The WHO recommends IPT for all HIV-positive pregnant women [38]. To date, it has not issued formal guidelines for the management of LTBI in HIV-negative pregnant women. In 2012, however, 60% of active TB diagnoses in women occurred in HIV-negative women [1]. Currently, the Centers for Disease Control and Prevention (CDC) recommend 6–9 months of IPT for pregnant women with a positive LTBI test, especially for high-risk women, including those from TB-endemic countries such as India [39]. No studies have directly examined the safety of INH in pregnancy [5]. INH may increase the risk of hepatotoxicity in pregnancy, though a decision-analysis study concluded that proper monitoring minimizes the risk [40]. INH crosses the placenta but does not seem to cause significant fetal toxicity. It is compatible with breastfeeding [41].

Our findings highlight the challenges of implementing TB prevention strategies in pregnant women. Choice and timing of LTBI screening during pregnancy have public health impacts: If 1,000 pregnant women were screened for LTBI using the TST at delivery, when their immune systems were maximally suppressed, just 110 of them would be diagnosed with and treated for LTBI. If, however, the same 1,000 women were screened with the QGIT postpartum, 520 of them—almost five-times as many—would be diagnosed and treated. With 180 million women becoming pregnant in high TB burden countries each year, choice of LTBI test and timing of LTBI screening matter and should be further evaluated in program settings.

## Supporting Information

Table S1 A) TST and QGIT results in antenatal clinic (ANC), n = 154, B) TST and QGIT results in delivery ward, n = 148, C) TST and QGIT results in immunization clinic, n = 99.
(DOCX)

Table S2 A) Effect of changing TST cutoff on concordance (ANC/Delivery/Postpartum combined, n = 351), B) Effect of changing QGIT cutoff on concordance (ANC/Delivery/Postpartum combined, n = 401).
(DOCX)

Table S3 Covariates associated with Positive TST and Positive QGIT results.
(DOCX)

Table S4 Proportion of TST versus QGIT results that transitioned from positive to negative result or negative to positive result in women from the longitudinal cohort.
(DOCX)
