# Supplementary material for: Pregnancy Differentially Impacts Performance of Latent Tuberculosis Diagnostics in a High-Burden Setting
Source: PLoS One. 2014 Mar 21;9(3):e92308. doi: 10.1371/journal.pone.0092308 (PMC3962385; doi:10.1371/journal.pone.0092308)
Supplement: Table S1 — A) TST and QGIT results in antenatal clinic (ANC), n = 154, B) TST and QGIT results in delivery ward, n = 148, C) TST and QGIT results in immunization clinic, n = 99. (DOCX) [file pone.0092308.s001.docx]

Supplemental Table S1a. TST and QGIT results in antenatal clinic (ANC), n=154

| Test Result | TST positive, n (%) | TST negative, n (%) | TST not read, n (%) |
| --- | --- | --- | --- |
| QGIT positive, n (%) | 20 (12%) | 25 (16%) | 5 (3.2%) |
| QGIT negative, n (%) | 5 (3.2%) | 85 (55%) | 9 (5.8%) |
| QGIT indeterminate, n (%) | 0 (0%) | 5 (3.2%) | 0 (0%) |

Supplemental Table S1b. TST and QGIT results in delivery ward, n=148

| Test Result | TST positive, n (%) | TST negative, n (%) | TST not read, n (%) |
| --- | --- | --- | --- |
| QGIT positive, n (%) | 13 (8.7%) | 33 (22%) | 2 (1.3%) |
| QGIT negative, n (%) | 2 (1.3%) | 90 (60%) | 4 (2.7%) |
| QGIT indeterminate, n (%) | 1 (0.67%) | 3 (2%) | 0 (0%) |

Supplemental Table S1c. TST and QGIT results in immunization clinic, n=99

| Test Result | TST positive,  n (%) | TST negative,  n (%) | TST not read,  n (%) |
| --- | --- | --- | --- |
| QGIT positive, n (%) | 13 (13%) | 21 (21%) | 18 (18%) |
| QGIT negative, n (%) | 5 (5%) | 31 (31%) | 11 (11%) |
| QGIT indeterminate, n (%) | 0 (0%) | 0 (0%) | 0 (0%) |

Abbreviations: TST indicates tuberculin skin test, QGIT indicates QuantiFERON®-TB Gold Test In-Tube
