# Supplementary material for: Pregnancy Differentially Impacts Performance of Latent Tuberculosis Diagnostics in a High-Burden Setting
Source: PLoS One. 2014 Mar 21;9(3):e92308. doi: 10.1371/journal.pone.0092308 (PMC3962385; doi:10.1371/journal.pone.0092308)
Supplement: Table S2 — A) Effect of changing TST cutoff on concordance (ANC/Delivery/Postpartum combined, n = 351), B) Effect of changing QGIT cutoff on concordance (ANC/Delivery/Postpartum combined, n = 401). (DOCX) [file pone.0092308.s002.docx]

Supplemental Table S2a. Effect of changing TST cutoff on concordance (ANC/Delivery/Postpartum combined, n=351)

| Results | TST Cutoff, mm | | |
| --- | --- | --- | --- |
|  | ≥5 | ≥10 | ≥15 |
| Positive TST/positive QGIT | 50 | 44 | 24 |
| Positive TST/negative QGIT | 18 | 11 | 5 |
| Negative TST/positive QGIT | 74 | 80 | 100 |
| Negative TST/negative QGIT | 201 | 208 | 214 |
| Agreement, % | 71% | 71% | 67% |
| κ (95% CI) | 0.33 (0.23-0.43) | 0.32 (0.23-0.42) | 0.19 (0.10-0.27) |

Supplemental Table S2b. Effect of changing QGIT cutoff on concordance (ANC/Delivery/Postpartum combined, n=401)

| Results | QGIT cutoff, IU/mL | | |
| --- | --- | --- | --- |
|  | ≥0.2 | ≥0.35 | ≥0.5 |
| Positive TST/positive QGIT | 48 | 47 | 46 |
| Positive TST/negative QGIT | 12 | 13 | 14 |
| Negative TST/positive QGIT | 88 | 79 | 71 |
| Negative TST/negative QGIT | 198 | 207 | 215 |
| Agreement, % | 69% | 71% | 73% |
| κ (95% CI) | 0.31 (0.21-0.40) | 0.32 (0.23-0.42) | 0.35 (0.25-0.45) |

Abbreviations: CI indicates confidence interval, TST indicates tuberculin skin test, QGIT indicates QuantiFERON®-TB Gold Test In-Tube
