# Supplementary material for: Pregnancy Differentially Impacts Performance of Latent Tuberculosis Diagnostics in a High-Burden Setting
Source: PLoS One. 2014 Mar 21;9(3):e92308. doi: 10.1371/journal.pone.0092308 (PMC3962385; doi:10.1371/journal.pone.0092308)
Supplement: Table S3 — Covariates associated with Positive TST and Positive QGIT results. (DOCX) [file pone.0092308.s003.docx]

Supplemental Table S3. Covariates associated with Positive TST and Positive QGIT results

| Covariate | TST positive (≥ 10mm) | | | QGIT positive (≥ 0.35 IU/mL) | | |
| --- | --- | --- | --- | --- | --- | --- |
|  | No. positive/ Total with results (%) | Unadjusted OR (95% CI, p) | Adjusted OR (95% CI, p) | No. positive/ Total with results (%) | Unadjusted OR (95% CI, p) | Adjusted OR (95% CI, p) |
| Stage of pregnancy |  |  |  |  |  |  |
| ANC | 26/143 (18%) | 1 | 1 | 50/154 (32%) | 1 | 1 |
| Delivery | 16/143 (11%) | 0.56 (0.28-1.1, p=0.09) | 0.60 (0.29-1.2, p= 0.16) | 48/148 (32%) | 0.99 (0.62-1.5, p=0.96) | 1.13 (0.68-1.8, p=0.62) |
| Postpartum | 18/70 (25%) | 1.5 (0.78-3.0, p=0.2) | 1.9 (0.93-3.9, p=0.07) | 52/99 (52%) | 2.03 (1.2-3.3, p=0.007) | 2.3 (1.3-3.9, p=0.002) |
| Place of residence |  |  |  |  |  |  |
| Rural | 4/36 (11%) | 1 | 1 | 8/37 (18%) | 1 | 1 |
| Urban/periurban | 56/320 (17%) | 1.6 (0.57-4.9, p=0.33) | 1.8 (0.50-6.6, p=0.36) | 143/358 (33%) | 2.0 (0.95-4.2, p=0.06) | 2.54 (1.0-5.9, p=0.03) |
| Employment |  |  |  |  |  |  |
| Unemployed | 57/333 (17%) | 1 | 1 | 138/376(36%) | 1 | 1 |
| Employed | 3/23 (13%) | 0.72 (0.20-2.5, p=0.61) | 0.63 (0.16-2.3, p=0.49) | 13/27 (48%) | 1.7 (0.78-3.7, p=0.17) | 1.7 (0.76-4.1, p=0.17) |
| Household members employed | |  |  |  |  |  |
| 1 person | 19/137 (13%) | 1 | 1 | 53/155 (34%) | 1 | 1 |
| ≥ 2 people | 41/219(18%) | 1.4 (0.79-2.5, p=0.23) | 1.5 (0.70-3.2, p=0.28) | 98/248 (37%) | 1.3 (0.86-1.9, p=0.19) | 1.4 (0.85-2.5, p=0.16) |
| Education |  |  |  |  |  |  |
| >4^th^ grade | 48/312 (15%) | 1 | 1 | 133/353 (37%) | 1 | 1 |
| ≤4^th^ grade | 12/43 (27%) | 2.1 (1.0-4.4, p=0.04) | 3.12 (1.4-6.8, p=0.005) | 18/49 (36%) | 0.96 (0.52-1.7, p= 0.9) | 1.2 (0.63-2.3, p=0.55) |
| Religion |  |  |  |  |  |  |
| Non-Muslim | 52/303 (17%) | 1 | 1 | 133/343 (38%) | 1 | 1 |
| Muslim | 8/53 (15%) | 0.85 (0.38-1.9, p=0.71) | 0.78 (0.33-1.8, p=0.57) | 18/60 (30%) | 0.67 (0.37-1.2, p=0.18) | 0.63 (0.34-1.1, p=0.14) |
| Cooking fuel |  |  |  |  |  |  |
| Gas/Kerosene | 55/319 (17%) | 1 | 1 | 140/362 (38%) | 1 | 1 |
| Biomass | 5/37 (13%) | 0.75 (0.27-2.0, p=0.56) |  | 10/40 (25%) | 0.81 (0.41-1.6, p=0.55) |  |
| Food Insecurity |  |  |  |  |  |  |
| Secure/Mild | 53/315 (16%) | 1 | 1 | 133/358 (37%) | 1 | 1 |
| Moderate/  Severe | 6/29 (20%) | 1.3 (0.51-3.3, p=0.57) |  | 13/34 (38%) | 1.2 (0.61-2.5, p=0.54) |  |
| TB symptom screen | |  |  |  |  |  |
| Negative | 56/323 (17%) | 1 | 1 | 136/368 (36%) | 1 | 1 |
| Positive | 3/28 (10%) | 0.57 (0.16-1.96, p=0.37) |  | 12/29 (41%) | 1.2 (0.59-2.7, p=0.52) |  |
| Household TB symptom screen | |  |  |  |  |  |
| Negative | 51/315 (16%) | 1 | 1 | 134/357 (37%) | 1 | 1 |
| Positive | 8/36 (22%) | 1.4 (0.63-3.4, p=0.36) |  | 15/41 (36%) | 0.87 (0.44-1.7, p=0.69) |  |
| Known TB contact |  |  |  |  |  |  |
| No | 59/344 (17%) | 1 | 1 | 144/390 (36%) | 1 | 1 |
| Yes | 0/9 (0%) | 1 | - | 5/10 (50%) | 1.5 (0.44-5.4, p=0.48) |  |
| Family type |  |  |  |  |  |  |
| Nuclear | 15/117 (12%) | 1 | 1 | 48/133 (36%) | 1 | 1 |
| Joint | 44/235 (18%) | 1.5 (0.83-2.9, p=0.16) | 1.3 (0.58-2.9, p=0.51) | 101/266 (37%) | 1.1 (0.72-1.7, p=0.61) | 0.9 (0.52-1.5,p=0.74) |
| Number of adults in home | - | 1.05 (0.97-1.1, p=0.20) |  | - | 1.03 (0.97-1.0, p=0.31) |  |
| Smoker |  |  |  |  |  |  |
| No | 46/279 (16%) | 1 | 1 | 117/315 (37%) | 1 | 1 |
| Yes | 11/60 (18%) | 1.1 (0.54-2.3, p=0.72) |  | 27/70 (38%) | 1.1 (0.70-2.0, p=0.52) |  |
|  |  |  |  |  |  |  |

Abbreviations: ANC indicates antenatal, CI indicates confidence interval, OR indicates odds ratio, QGIT indicates QuantiFERON®-TB Gold Test In-Tube, TST indicates tuberculin skin test
