# Supplementary material for: Pregnancy Differentially Impacts Performance of Latent Tuberculosis Diagnostics in a High-Burden Setting
Source: PLoS One. 2014 Mar 21;9(3):e92308. doi: 10.1371/journal.pone.0092308 (PMC3962385; doi:10.1371/journal.pone.0092308)
Supplement: Table S4 — Proportion of TST versus QGIT results that transitioned from positive to negative result or negative to positive result in women from the longitudinal cohort. (DOCX) [file pone.0092308.s004.docx]

Supplemental Table S4: Proportion of TST versus QGIT results that transitioned from positive to negative result or negative to positive result in women from the longitudinal cohort

| **Stage of Pregnancy** | **TST** | **QGIT** | **p value** |
| --- | --- | --- | --- |
| **Antepartum to Delivery** | N=41^a^ | N=60 |  |
| -Negative to Positive | 2/33 (6%) | 5/43 (11%) | 0.69 |
| -Positive to Negative | 5/8 (62%) | 3/17 (17%) | 0.06 |
| **Delivery to Postpartum** | N=11^b^ | N=15 |  |
| -Negative to Positive | 2/10 (20%) | 5/10 (50%) | 0.35 |
| -Positive to Negative | 1/1 (100%) | 2/5 (40%) | >0.95 |

^a^ Only 41 out of the 60 women returned for TST reading at delivery

^b^ Only 11 out of 15 women had TST reading done at delivery and postpartum
